# Acute Transverse Myelitis Associated with Buserelin Use during IVF

**DOI:** 10.1155/2013/386765

**Published:** 2013-03-28

**Authors:** Djavid I. Alleemudder, Khaled Sadek, Shaun Fountain, Sallie Davies

**Affiliations:** ^1^Salisbury District Hospital, Salisbury SP2 8BJ, UK; ^2^Southampton General Hospital, Southampton SO16 6YD, UK

## Abstract

A healthy woman undergoing in vitro fertilization (IVF) developed acute transverse myelitis (ATM) following the use of Buserelin. ATM has a multifactorial etiology and may develop as a result of the activation of immune responses. Infectious agents have been postulated as possible triggers of an immune response (Sá, 2009). Gonadotropin-releasing agonists may have a similar role and trigger the acceleration of preexisting disease by the activation of immune responses (Ho et al., 1995, and Umesaki et al., 1999).

## 1. Introduction

Buserelin is a gonadotropin-releasing hormone agonist administered subcutaneously to downregulate pituitary release of luteinising hormone (LH) and follicular stimulating hormone (FSH). It has been used extensively in gynaecology, particularly in assisted conception to suppress pituitary function prior to follicular recruitment [1]. 

We report on an unusual presentation of acute transverse myelitis (ATM) following the administration of Buserelin during in vitro fertilization (IVF). 

## 2. Case Report

A 34-year-old Caucasian woman presented to the fertility clinic with a 3-year history of secondary infertility. She had undergone an emergency caesarean section for placental abruption 4 years prior and had subsequently made a full recovery. Her menstrual cycle was regular and normal. Initial investigations focused on possible causes for the secondary infertility. The female hormonal profile was normal for ovarian reserve (FSH 5.5, LH 2.0, oestradiol 262), thyroid function, antiphospholipid syndrome, prolactin, and androgen profile. A transvaginal pelvic ultrasound scan identified an arcuate uterus with normal ovaries and no other pelvic pathology. A hysterosalpingogram confirmed bilateral tubal patency without any evidence of intrauterine synechiae. Her medical and gynaecological background was otherwise unremarkable. Incidentally, there was no contributing male factor to explain the cause of the infertility. She was commenced on Buserelin on day 21 of her menstrual cycle as part of an agonist cycle IVF.

**Figure 1:**
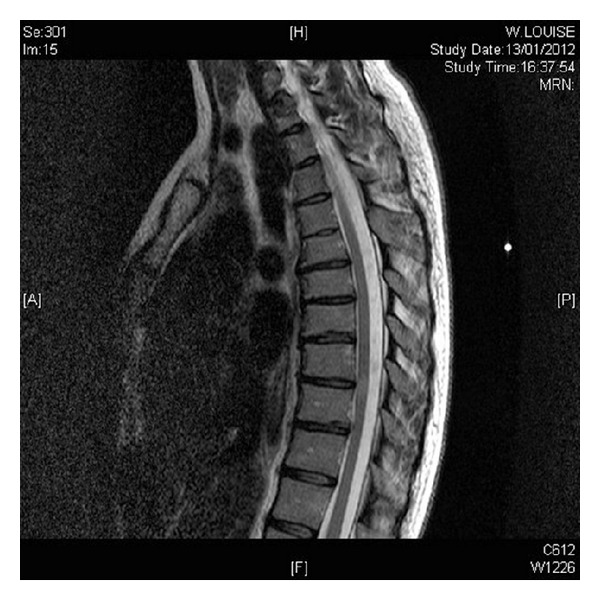
Sagittal view showing increased signal seen at the level of C5 and T8/8, appearances compatible with an inflammatory cause.

**Figure 2:**
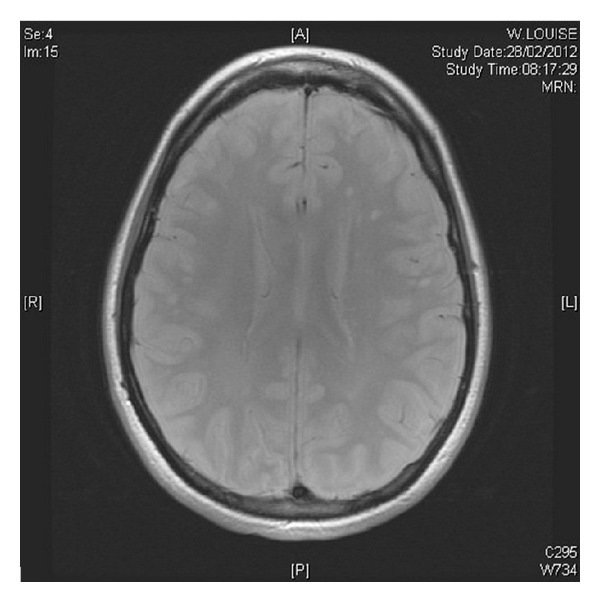
Axial view showing periventricular lesions supporting a clinical diagnosis of demyelination in the clinical context and knowledge of the spine.

**Figure 3:**
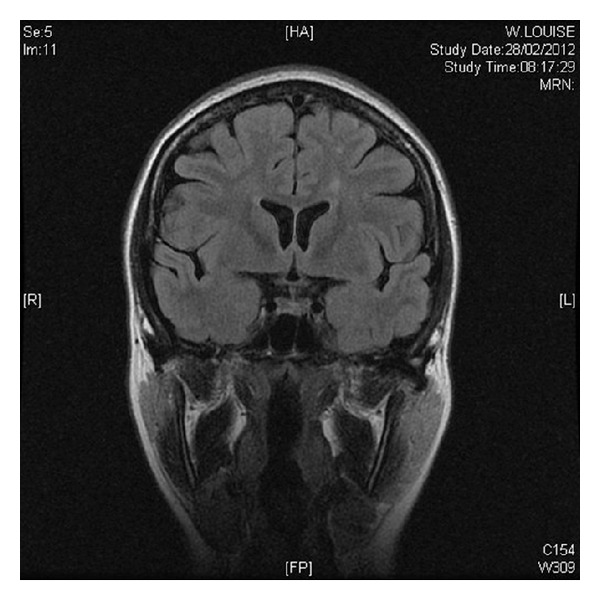
Coronal view showing periventricular lesions supporting a clinical diagnosis of demyelination in the clinical context and knowledge of the spine.

After 12 days use of Buserelin, the patient noted bilateral ascending paraesthesia in the lower extremities. There was no prior history of any neurological deficit and no additional neurological symptoms noted. Magnetic resonance imaging identified 2 lesions within the spinal cord and several lesions within the brain matter (Figures 1, 2 and 3). A diagnosis of acute transverse myelitis was made and multiple sclerosis is considered as a possible differential.

The patient was given a short course of intravenous corticosteroids and made a full recovery without any residual symptoms. She recommenced IVF treatment using a gonadotropin-releasing hormone antagonist to avoid any possible reoccurrence of her symptoms with Buserelin.

## 3. Discussion

Acute transverse myelitis (ATM) falls within the spectrum of acute myelopathies. There is an acute or subacute inflammation of the spinal cord, which manifests as bilateral weakness, sensory loss, paraesthesia, and autonomic dysfunction. It has a reported incidence of 4.6/million in the United States [2]. ATM has a heterogenous etiology including infectious, neoplastic, paraneoplastic, compressive, traumatic, demyelinating, immunological, and degenerative causes. The cause remains unknown in 21% of cases [2], although this number may represent an underestimation.

In 2002, the Transverse Myelitis Consortium Working Group (TMCWG) set out the inclusion criteria for idiopathic ATM which includes (1) the development of sensory, motor, and autonomic dysfunction attributable to the spinal cord; (2) bilateral neurological symptoms and signs; (3) a clearly defined sensory level; (4) symptoms worsening to a maximum over 4 hours–21 days; (5) exclusion of extra-axial compression by neuroimaging; (6) inflammation of the spinal cord by CSF analysis of IgG, pleocytosis, or gadolinium enhancement [3]. The presence of all of the inclusion criteria allows a definite diagnosis of idiopathic ATM.

The treatment of ATM requires the prompt administration of high dose corticosteroids to reduce the inflammatory process. Plasma exchange is warranted for those with severe demyelination (i.e., an inability to walk, marked autonomic dysfunction, sensory loss in the lower extremities, and minimal clinical improvement with intravenous steroids). Long-term rehabilitation programs improve functional capacity and mobility. The prognosis appears promising for the young, when acute/subacute progression occurs over days/weeks and if the posterior column and deep tendon reflexes are maintained. A third of patients will achieve full recovery from ATM usually within 3–6 months [4], whilst others will have residual symptoms or show no improvement following treatment [5].

 The adverse effects of Buserelin are well documented [6]. These include hypooestrogenic symptoms such as hot flushes and night sweats, vaginal dryness, loss of libido, headache, hypersensitivity reactions, reversible loss of bone mineral density, visual disturbances, arthralgia, depression, and emotional lability, but acute transverse myelitis has not yet been documented.

We do not believe that Buserelin directly caused ATM but its use may have acceerated the signs of preexisting disease due to transient effects on the immune system.

ATM may develop as a result of the activation of autoimmune responses. Certainly, there are clear links between ATM and systemic immune disorders such as systemic lupus erythematosus (SLE), sarcoidosis, antiphospholipid, Sjögren's and Behçet's syndromes [7]. Possible mechanisms postulated include (1) an acceleration of a preexisting autoimmune process (2) via the activation of B or T cells, resulting in humoral or cell-mediated derangements targeting the central nervous system. Microbial agents may pose as potential candidates to trigger an immune reaction to damage neural tissue. Indeed, an infectious state is often noted prior to the onset of ATM (between 25%–44% of cases). It is believed that these microbes could do this by activating the immune response directly, or indirectly by infecting a remote site, thereby activating a systemic immune response [3].

Similarly, GnRHa could also play an important role to trigger an immune response and initiate the development of ATM. Research concerning the immunomodulatory effects of GnRH agonists, however, is limited [8]. Ho et al. [9] investigated the effect of Buserelin on in vivo human immune cells and whether this alteration affected the success of pregnancy during IVF. The use of a GnRH agonist had a transiently immunosuppressive effect on CD4+ and CD25+ cells but the activity of CD69+ and HLA-DR+ T cells was potentiated during and after implantation [9]. Similarly, Umesaki et al. [10] noted an increase in natural killer (NK) cell activity following treatment with Buserelin in patients with endometriosis and uterine fibroids [10]. It therefore seems plausible that Buserelin could influence the development of ATM by way of its immunological effects.

This is the first paper highlighting a possible link between GnRH agonists and acceleration of signs of preexisting ATM. It is important to exclude organic central nervous system disease in patients complaining of neurological symptoms whilst using GnRH agonists. Further research is needed to unravel the potential immunomodulatory role of GnRH agonists and its association with the development of neurological conditions such as ATM.
